# Can Pets Replace Children? The Interaction Effect of Pet Attachment and Subjective Socioeconomic Status on Fertility Intention

**DOI:** 10.3390/ijerph18168610

**Published:** 2021-08-15

**Authors:** Zhen Guo, Xiaoxing Ren, Jinzhe Zhao, Liying Jiao, Yan Xu

**Affiliations:** Beijing Key Laboratory of Applied Experimental Psychology, National Demonstration Center for Experimental Psychology Education (Beijing Normal University), Faculty of Psychology, Beijing Normal University, Beijing 100875, China; guozhen@mail.bnu.edu.cn (Z.G.); 201828061152@mail.bnu.edu.cn (X.R.); 202031061025@mail.bnu.edu.cn (J.Z.); jiaoliying@mail.bnu.edu.cn (L.J.)

**Keywords:** pet attachment, subjective socioeconomic status, fertility intention

## Abstract

A growing number of young people tend to regard their pets as their surrogate children, yet research examining the relationship between pet attachment and fertility intention remains scarce. Moreover, individuals’ fertility intention is affected by economic resources. Therefore, we conducted two studies to examine the interaction effect of pet attachment and subjective socioeconomic status (SES) on childbearing-aged individuals’ fertility intention. In Study 1, we utilized questionnaires to measure Chinese pet owners’ pet attachment, subjective SES, and fertility intention. In Study 2, participants’ pet attachment was experimentally manipulated by reading articles about the benefits of petkeeping. The results of the two studies consistently demonstrated that the effect of pet attachment on fertility intention was moderated by subjective SES. Specifically, pet attachment was negatively associated with fertility intention when individuals had a high level of subjective SES, whereas this effect disappeared when individuals had low subjective SES. These findings suggest an explanation for why individuals with high subjective SES delay or even opt out of childbearing. The limitations and implications of the current study are discussed.

## 1. Introduction

Currently, many countries face challenges of low fertility rates. In China, fertility rates have begun to drastically plunge since the 1970s, and the total fertility rate has been well below the replacement level [1,2]. However, as the total fertility rate has declined in China, the number of pet owners has increased rapidly [3]. In 2019, 61.2 million Chinese residents kept a total of 99.15 million pets (dog or cat), an increase of 8.4% from 2018 [4]. The media in Japan and Mexico also suggested similar trends have developed as views toward animals as pets change alongside the rising consumerism in relation to pets and declining fertility [5,6].

As companion animals, pets can satisfy people’s psychological needs for companionship, friendship, and unconditional love. Hence, an increasing number of people treat pets as their family members [7]. Research has also revealed that childfree families tend to consider their dogs or cats to be similar to human children [8]. However, few studies have directly investigated the effect of pet attachment on childbearing-aged individuals’ fertility intention. Therefore, the current research aims to explore the relationship between pet attachment and individuals’ fertility intention. Moreover, individuals’ fertility intention is shaped by their own economic resources [9]. Specifically, previous research emphasized the importance of economic factors (e.g., income, occupation, and education level) in determining individuals’ fertility intention [10]. Therefore, we propose that pet attachment and subjective socioeconomic status (SES) might have an interaction effect on individuals’ fertility intention. Below, we discuss our theoretical rationale in detail.

A large number of studies have recognized the psychological, physical, and social benefits of human–animal interaction, especially for dog or cat owners [11,12,13]. Keeping pets is robustly associated with multiple aspects of psychological well-being, including higher levels of positive affect, happiness, and self-esteem [14] and lower levels of depression, loneliness, and perceived stress [15]. In addition, studies have demonstrated that petkeeping can benefit individuals’ physical health. For example, research suggests that pets can motivate people to exercise, which is linked to better health [16]. Indeed, simply being in a room with a friendly dog can lower an individual’s blood pressure [17]. Additionally, having pets can provide links or bridges to other potential friends [18], which is beneficial to individual social function.

One of the most important reasons why pets have beneficial effects on individuals is that pet owners view pets as their children, and they have a strong pet attachment with them. Pet attachment refers to the intimate and lasting emotional connection between pets and humans [19,20]. Bowlby’s attachment theory [21] originally described the relationship between children and their parents. The main feature of parents’ attachment to their children is caregiving or protecting, which is similar to the feelings of pet owners toward their pets. Just as people can develop an intimate emotional connection with their children, they can also have strong emotional bonds with their pets. The patterns of attachment behaviors between pets and pet owners are consistent with those of infants and their parents [22]. For example, previous studies have found that humans are sensitive to the baby schema effect of animals, which serves as an innate releasing mechanism in adults to protect and nurture them [23]. One study also found that people talk to pets in motherese, which is similar to how parents talk to their infants [24]. Indeed, pet owners tend to regard their pets as substitute family members [25] or even as their children [26]. Thus, it is reasonable and imperative to investigate how pet attachment influences childbearing-aged individuals’ fertility intention.

Previous studies have found that people with higher pet attachment tend to regard their pets as their children [23,27]. However, very little attention has been paid to examining the relationship between pet attachment and fertility intention. On the one hand, petkeeping can evoke people’s desire to care and nature. Previous research has suggested that young adults who are more likely to attach to their pets express higher concern for the next generation [28]. Thus, individuals high in pet attachment might be more likely to have high childbearing intentions.

On the other hand, some studies have suggested that pets can replace children. A qualitative study of 12 childfree companion animal owners between the ages of 18 and 44 suggested that the participants tended to frame their pets as not just family members but as surrogate children and that the human–pet relationship could make them delay or opt out of childbearing [29]. Researchers have also suggested that young adults tend to keep pets instead of becoming parents [12]. Therefore, pet attachment might also be negatively associated with fertility intention. Given that the results of the relationship between pet attachment and fertility intention are inconsistent, the current study aims to address this gap.

However, the association between pet attachment and fertility intention might be different for childbearing-aged individuals with different levels of subjective SES. Subjective SES represents the perception of one’s own socioeconomic position or rank relative to that of others in the social class hierarchy [30,31]. Subjective SES is correlated with individuals’ objective SES (e.g., education, income), but it also contributes to social thoughts, emotion, and behavior independently of an individual’s objective resources [32]. As subjective SES is often ascribed subjective meaning that is influenced by the situational or broader social context, such as other individuals to whom an individual compares his or her income or educational level [33], the subjective perception of social class has more important insights than objective SES into psychological processes, such as self-perceptions [34], system justification belief [35], and subjective well-being [31].

The psychological orientation approach to SES suggests that perceptions of one’s social class rank produce different characteristic patterns of thought, feelings, and behaviors [36,37]. Individuals with a higher level of subjective SES take for granted the resources and opportunities they have; thus, they tend to have a self-focus and expect to maintain control, neglecting the role of contextual constraints on their intention and behavior. In contrast, the life outcomes of individuals with lower subjective class are often under the control of outside forces; therefore, they develop higher sensitivity to the social context and interdependence with the individuals within this context [38]. Regarding the fertility choices of individuals with different levels of subjective SES, it can be inferred that the fertility intention of individuals with lower subjective SES will be more influenced by the social context rather than their own interests or wishes. Their fertility intention will be more likely to be influenced by their parents and to be consistent with societal expectations. Therefore, they might not be affected by their emotional connections with their pets. In contrast, the fertility choices of individuals with higher subjective SES are more likely to be driven by their internal states, personal goals, and emotions [37]. When these individuals make fertility choices, they might be influenced by the level of pet attachment. Thus, we infer that subjective SES and pet attachment have interaction effects on individuals’ fertility intention. Specifically, the fertility choices of individuals with high subjective SES might be associated with pet attachment, while the fertility choices of individuals with low subjective SES will not be affected by pet attachment.

**Figure 1:**
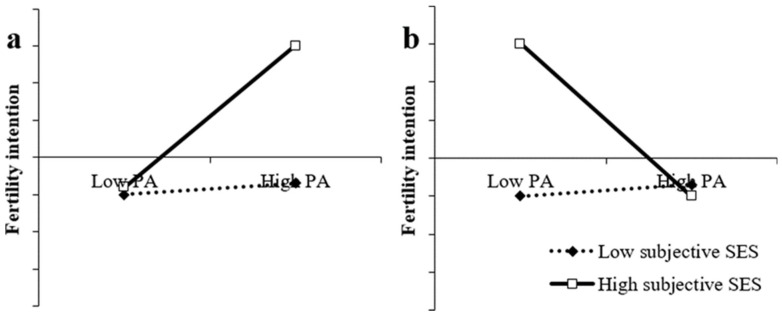
Model of the hypothetical pet attachment × subjective SES interaction depicting (**a**) an interaction in which pet attachment promotes fertility intention in individuals with high subjective SES but a nonsignificant relationship between pet attachment and fertility intention in individuals with low subjective SES; (**b**) an interaction in which pet attachment inhibits fertility intention in individuals with high subjective SES but a nonsignificant relationship between pet attachment and fertility intention in individuals with low subjective SES.

Overall, we propose two hypotheses: (a) for individuals with high levels of subjective SES, pet attachment is positively associated with fertility intention, while the fertility choices of individuals with low subjective SES are not influenced by pet attachment (Hypothesis 1a; see Figure 1a); (b) for individuals with high levels of subjective SES, pet attachment is negatively associated with fertility intention, while the fertility choices of individuals with low subjective SES are not influenced by pet attachment (Hypothesis 1b; see Figure 1b).

## 2. The Current Study

We conducted two studies to examine the interaction effect of pet attachment and subjective SES on childbearing-aged individuals’ fertility intention. In Study 1, we utilized questionnaires to measure Chinese pet owners’ pet attachment, subjective SES, fertility intention, and other control variables to examine the interaction effect of pet attachment and subjective SES on fertility intention. In Study 2, both pet-owners and non-pet owners were recruited. We manipulated the participants’ pet attachment and then measured their subjective SES and fertility intention. Previous research has suggested that nearly 10% of first births occur to women over the age of 35 years old, whereas 21% of first births occur to women under age 20 [39]. Most women have their first child between the ages of 20 and 35. Thus, we recruited male and female participants aged between 20 and 35.

## 3. Study 1

### 3.1. Methods

#### 3.1.1. Participants

As we were unsure of the appropriate sample size, we recruited as many participants as our resources permitted prior to any data analysis. We recruited childbearing-aged (age range: 20–35 years) participants by distributing survey links through university online forums and social media, such as WeChat. A total of 162 Chinese pet owners completed the survey on a Chinese survey website (https://www.wjx.cn (accessed on 5 April 2019)) for CN¥5 (CN¥1 = USD 0.15). Seven participants were excluded from the data screening process because they chose the same option on all items. The valid sample in Study 1 consisted of 155 Chinese adults (27.1% men; mean age = 27.41 years, *SD* = 3.82). All the research procedures met the ethical guidelines of the American Psychological Association and were approved by the Academic Ethics Committee of the Faculty of Psychology at Beijing Normal University (20181220).

#### 3.1.2. Materials and Procedure

The participants were told to complete a questionnaire regarding their living conditions as pet owners. The questionnaire was organized as follows. First, all participants completed the Lexington Attachment to Pets Scale. Second, the subjects completed measures of fertility intention. Next, all participants completed the MacArthur Scale of Subjective Status and other demographic questions indicating their age, gender, marital status, education level, and whether they had children.

Pet attachment. The participants completed the 23-item Lexington Attachment to Pets Scale (LAPS) on a 4-point scale (1 = strongly disagree, 4 = strongly agree) [19]. This questionnaire includes questions regarding the quality of one’s relationship with his or her pet (e.g., I think my pet is just a pet, and I feel that my pet is part of the family). The Cronbach’s alpha with the current sample was 0.92.

Fertility intention. We measured the participants’ fertility intention with a four-item scale. This scale was adapted from previous studies (see Appendix A for all items) [40]. The participants rated each item on a 9-point Likert scale ranging from 1 (strongly disagree) to 9 (strongly agree). The Cronbach’s alpha coefficient was 0.87.

Subjective SES. The participants’ subjective SES was measured by the MacArthur Scale of Subjective Social Status [30]. They were shown a picture of a 10-rung ladder representing social status and were asked to indicate their position on the ladder (1 = the lowest, 10 = the highest).

Control variables. We measured the participants’ gender, age, educational level, marital status, and the number of children (if any) as control variables. The participants reported their education level (1 = high school or lower, 2 = associate degree, 3 = bachelor’s degree, 4 = master’s degree or higher) on a 4-point scale. The participants’ marriage status was coded into three categories (1 = single, 2 = in love, 3 = married). The participants also reported whether they had children (0 = no children, 1 = have children).

### 3.2. Results and Discussion

The descriptive statistics and bivariate correlations are shown in Table 1. Subjective SES did not significantly correlate with pet attachment or fertility intention. Moreover, pet attachment was not correlated with fertility intention.

Next, we used hierarchical regression to test the hypothesized interaction between subjective SES and pet attachment on fertility intention. To test our hypotheses, we entered the control variables in Model 1 of the regression. In Model 2, we entered the main effects of subjective SES and pet attachment after standardizing them as *Z* scores. In Model 3, after standardizing the variables, we entered the interaction term (subjective SES × pet attachment). The results of the regression analyses showed that gender and children significantly influenced fertility intention. Specifically, women had lower fertility intention. Those who already had children tended to have higher fertility intention. Neither subjective SES nor pet attachment predicted fertility intention (*∆R*^2^ = 0.013, *p* = 0.31). As shown in Table 2 and Table 3, the interaction between pet attachment and subjective SES significantly predicted fertility intention beyond the controls and the main effects of subjective SES and pet attachment (*∆R*^2^ = 0.036, *p* = 0.009).

**Figure 2:**
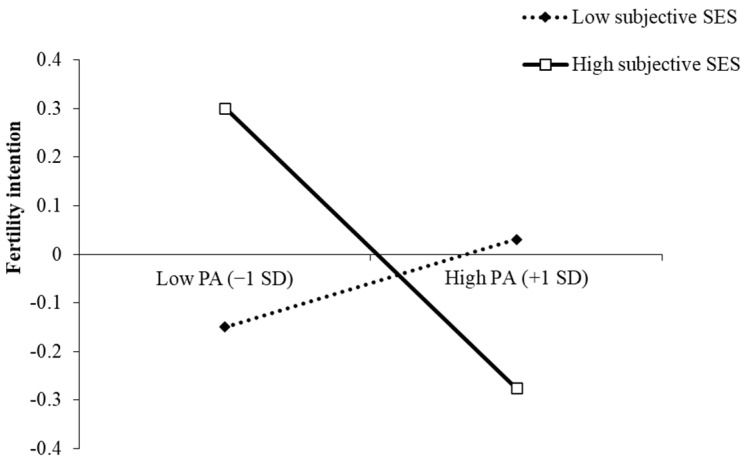
Interaction between subjective SES and pet attachment on fertility intention.

We further conducted simple slope analysis and plotted the results for low (mean − 1 SD) and high (mean + 1 SD) levels of subjective SES in Figure 2. Among the participants with high subjective SES, higher pet attachment was significantly associated with lower fertility intention, *B_simple_* = −0.29, *SE* = 0.10, *t* = −2.88, *p* = 0.004. For the participants with low subjective SES, the relationship between pet attachment and fertility intention was non-significant, *B_simple_* = 0.09, *SE* = 0.11, *t* = 0.84, *p* = 0.40. Thus, Hypothesis 1b was supported.

Study 1 provided preliminary support for our hypotheses regarding the interaction effect of subjective SES and pet attachment on pet owners’ fertility intention. However, whether the interactive role of subjective SES and pet attachment would also occur in individuals who do not keep pets in their daily lives was still unknown. Thus, Study 2 aimed to replicate and extend the present findings in a childbearing-aged sample who were not required to keep pets to be included in the sample. Moreover, in Study 2, we manipulated the participants’ pet attachment to establish the model of the interaction effect of subjective SES and pet attachment on fertility intention.

## 4. Study 2 

Study 2 used a two-condition, between-subjects experimental design in which we manipulated the participants’ pet attachment (high pet attachment vs. control) to replicate the interactive role of subjective SES and pet attachment on fertility intention in a sample with both pet owners and non-pet owners.

### 4.1. Methods

#### 4.1.1. Participants

A priori power analysis using G*Power software [41] with the interaction effect size from Study 1 (f = 0.1937), α = 0.05, and power = 0.80 indicated the necessary total sample size to be 212. To exceed this sample size requirement, we recruited 255 childbearing-aged (20–35 years old) participants via a reliable online Chinese data collection platform that is similar to Qualtrics Online Sample (https://www.wjx.cn (accessed on 14 May 2019)). A total of 255 participants completed the survey for CN¥5. Nine participants were excluded because they did not comply with the manipulation as instructed. Twelve participants were excluded because they chose the same option on all items. The valid sample in Study 2 consisted of 234 Chinese adults (38.5% men; mean age = 25.38 years, *SD* = 3.74). All the research procedures met the ethical guidelines of the American Psychological Association and were approved by the Academic Ethics Committee of the Faculty of Psychology at Beijing Normal University (20181220).

#### 4.1.2. Materials and Procedure

On the first page of the questionnaire, all participants read that the purpose of the study was to examine people’s attitudes toward pets. After giving their informed consent, the participants were randomly assigned to one of the two pet attachment conditions: a high-attachment condition (*n* = 121) or a control condition (*n* = 113). Then, the participants’ fertility intention was assessed with the same item as in Study 1. Next, all participants completed the MacArthur Scale of Subjective Social Status and other demographic questions indicating their age, gender, marital status, education level, and whether they had a child, as in Study 1. In addition, we asked the participants to indicate their monthly income (1 = lower than CN¥2000, 2 = CN¥2000–CN¥5000, 3 = CN¥5000–CN¥10,000, 4 = CN¥10,000–CN¥20,000, 5 = greater than CN¥20,000; *M* = 2.75, *SD* = 0.69) and whether they had pets (0 = no pets, 1 = have pets). We standardized monthly income and educational level and then summed the two scores as a composite measure of objective SES [42].

Manipulation of pet attachment. In the high-attachment condition, the participants were required to read an article about the benefits of keeping a pet. For example, one paragraph stated, “Pets not only provide valuable companionship for people but also make pet owners feel needed. Caring for an animal can reduce stress, anxiety, ease loneliness, and improve one’s subjective well-being. Giving unconditional love to pets gives meaning to some people’s lives.” After the participants read this article, we presented them with some pictures of people enjoying being with their pets. The participants in the control condition were asked to recall a recent shopping experience.

As a manipulation check, the participants completed the modified 6-item LAPS (e.g., owning a pet can add to happiness) [19]. Considering the length of the questionnaire, we only used six items of the original scale to check the manipulation effect. The participants rated each item on a 4-point scale (1 = strongly disagree, 4 = strongly agree). The Cronbach’s alpha coefficient was 0.79.

### 4.2. Results and Discussion

First, we examined the manipulation effect. An independent-samples *t*-test revealed that the participants in the high attachment condition were more likely to have a higher level of pet attachment (*M* = 3.29, *SD* = 0.44) than those in the control condition (*M* = 3.08, *SD* = 0.60); *t* (232) = 2.94, *p* = 0.004, *d* = 0.38.

Next, we tested the interaction effect of pet attachment (0 = control condition, 1 = high attachment) and subjective SES on fertility intention using hierarchical regression. Prior to the analyses, the predictors were all standardized as *Z* scores. First, we entered the control variables in Model 1 of the regression. In Model 2, we entered the main effects of subjective SES and pet attachment. In Model 3, we entered the interaction term (subjective SES × pet attachment). The results of the regression analyses showed that gender significantly influenced fertility intention. Women tended to have lower fertility intention. Neither subjective SES nor pet attachment predicted fertility intention (*∆R*^2^ = 0.017, *p* = 0.12). As shown in Table 4 and Table 5, pet attachment and subjective SES had a marginally significant interaction effect on fertility intention beyond the controls and the main effects of subjective SES and pet attachment (*∆R*^2^ = 0.014, *p* = 0.056).

**Figure 3:**
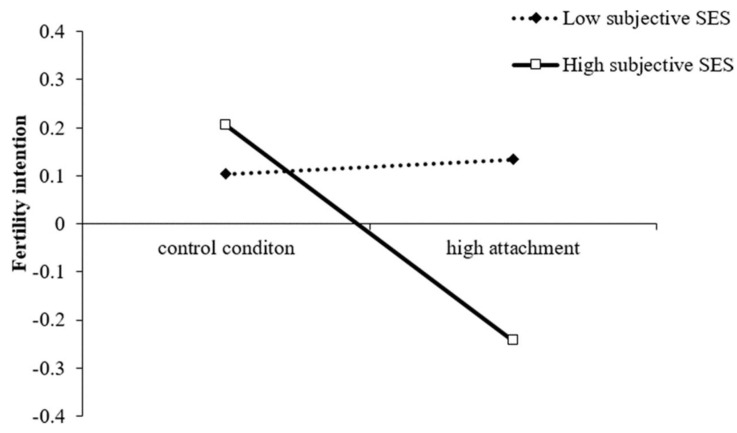
Interaction between subjective SES and pet attachment on fertility intention.

We further conducted simple slope analysis and plotted the results for low (mean − 1 *SD*) and high (mean + 1 *SD*) levels of subjective SES in Figure 3. Among the participants with low subjective SES, pet attachment was not significantly associated with their fertility intention, *B_simple_* = 0.03, *SE* = 0.17, *t* = 0.17, *p* = 0.86. For the participants with high subjective SES, the negative relationship between pet attachment and fertility intention was significant, *B_simple_* = −0.45, *SE* = 0.17, *t* = −2.57, *p = 0*.01. Thus, Hypothesis 1b was supported.

## 5. Discussion

In the two studies, we examined the interaction effect of pet attachment and subjective SES on fertility intention among a Chinese pet owner sample and a sample of the adult population. We found that neither subjective SES nor pet attachment was significantly associated with fertility intention. However, pet attachment and subjective SES had a significant interaction effect on fertility intention. Specifically, in support of Hypothesis 1b, pet attachment was negatively associated with fertility intention when individuals had high subjective SES, whereas pet attachment was not significantly associated with fertility intention when individuals had low subjective SES.

These findings indicated that the role of individuals’ subjective SES helps to understand the relationship between pet attachment and fertility intention. Unlike a previous qualitative study that suggested that when people regard their pets as surrogate children, they are more likely to have low level of fertility intention [29], our results revealed that the negative relationship between pet attachment and fertility intention existed only in people with a high level of subjective SES. When individuals feel they have a relatively higher SES than others, they are more likely to make fertility decisions according to their own wishes and interests regardless of situational constraints [37]. Thus, when people have a stronger attachment to their pets, they may consider them as their children to satisfy their emotional needs, which makes them not feel the need to have children [27]. Another reason for the negative relationship between pet attachment and fertility intention among people high in subjective SES is that they might choose to be childfree first and then develop an emotional bond with their pets to fulfill their need to nurture. A previous study also found that more educated women are more likely to use contraception and delay childbearing [43]. Although Study 2 experimentally manipulated the participants’ pet attachment, the causal relationship between pet attachment and fertility intention still needs to be clarified in future studies using a longitudinal design. In addition, besides the emphasis on the importance of pet attachment and subjective SES in shaping individuals’ fertility intention, the current study does not rule out the possibility of genetic or biological influence. Women’s biological mechanisms can also be linked with expectations of childlessness [44]. More research is needed to better understand the interaction between pet attachment, subjective SES, biological mechanisms, and the impact on fertility intention.

Moreover, we found that pet attachment was not associated with the fertility intention of individuals with low subjective SES. When people believe that they have lower SES than others, many other external factors, such as the cost of raising children or their parents’ expectations instead of their own internal states (i.e., pet attachment), may influence their fertility decisions [36]. Moreover, as a pronatalist society, social norms in the traditional Chinese context encourage people to have more children. Expressions such as “more sons, more happiness” (duo zi duo fu) and “life is more complete with both a son and a daughter” (er nü shuang quan) reflect this cultural tradition. Compared to individuals with high subjective SES, people with low subjective SES tend to have fewer psychosocial resources and feel more pressured by their life circumstances [45]. Thus, they are less likely to be affected by pet attachment and to challenge the social norms to have fewer children. Therefore, their fertility intention might be more influenced by their parents, friends, or social norms instead of their pet attachment.

The present research makes a significant contribution to the literature on how pet attachment influences individuals’ fertility intention. There have been few empirical studies investigating the relationship between pet attachment and fertility intention. More importantly, there have been inconsistent results regarding this relationship. On the one hand, some studies have suggested that strong pet attachment leads people to have a stronger motivation to rear children [28]. On the other hand, some research has suggested that having pets make people have low fertility intention [29]. Our results indicated that people with high subjective SES who feel a strong bond to the pets were more likely to regard their pets as their children, which made them have low fertility intention. Such individuals’ strong attachment to their pets may reinforce a delay in childbearing or their decision not to have children by satisfying the need to nurture.

Several limitations of the present study should also be noted. First, the current research examined the interaction effect of pet attachment and subjective SES on individuals’ fertility intention. A previous study found that actual fertility behavior failed to match reported intention at the individual level [46]. Future research could measure people’s actual fertility behavior or use a large dataset to explore the relationship between pet consumption and fertility rate [9]. Second, individuals’ fertility choices might be culturally dependent, so more research is needed in cultural contexts beyond China. Third, although we experimentally manipulated the participants’ pet attachment, it is not appropriate to rule out the possibility that fertility intention could influence individuals’ pet attachment given the cross-sectional and correlational nature of the present study. The bidirectional relationship between pet attachment and fertility intention in people with a high level of subjective SES should be examined in future research. Fourth, participants in study 1 were recruited through the university online forums. Therefore, participants in study 1 are more likely to have higher SES and higher levels of education, which limited the generalizability of the findings. It is important for future studies to collect more diverse and representative samples.

Despite these limitations, the study is important in light of the increasing childfree trend in China [1]. Strong attachment to pets may well serve to increase the age at first childbirth or even provide the opportunity to decide to opt out of childbearing. As childlessness is becoming more common in China, our result somehow provides a new insight that pet attachment and subjective SES might interactively determine individuals’ fertility decision-making.

## Figures and Tables

**Table 1:** Descriptive statistics and bivariate correlations of the key variables.

Variables	*M*	*SD*	1	2	3	4	5	6	7	8
1 Age	27.41	3.82	1							
2 Gender	0.73	0.45	−0.02	1						
3 Marital status	2.05	0.84	0.46 ***	0.01	1					
4 Children	0.18	0.39	0.51 ***	−0.09	0.53 ***	1				
5 Education	3.14	0.84	−0.16 *	0.12	−0.07	−0.44 ***	1			
6 Subjective SES	5.17	1.48	0.12	−0.04	0.04	−0.04	0.18 *	1		
7 PA	3.24	0.43	0.06	0.14	0.01	−0.01	−0.20 *	−0.11	1	
8 FI	5.99	1.92	0.13	−0.23 **	0.17 *	0.37 ***	−0.21 **	0.01	−0.13	1

Note: Gender was a dummy variable coded as 0 = male and 1 = female. * *p* < 0.05, ** *p* < 0.01, *** *p* < 0.001. PA = pet attachment, FI = fertility intention.

**Table 2:** Summary of the hierarchical multiple regression (*N* = 155).

Model	R	R Square	R Square Change	F Change	Sig. F Change
1	0.431	0.186	0.186	6.800	0.001
2	0.446	0.199	0.013	1.182	0.310
3	0.485	0.235	0.036	6.899	0.009

**Table 3:** Summary of the hierarchical regression model coefficients (*N* = 155).

	Standardized (β)	Std. Error	95% Confidence Interval		*p*
			Low	High	
Model 1					
Age	−0.083	0.098	−0.276	0.110	0.399
Gender	−0.435	0.165	−0.762	−0.108	0.009
Marriage	−0.004	0.091	−0.184	0.176	0.964
Children	0.974	0.268	0.444	1.504	0.001
Education	−0.039	0.087	−0.210	0.133	0.657
Model 2					
Age	−0.075	0.099	−0.271	0.121	0.449
Gender	−0.392	0.168	−0.723	−0.061	0.021
Marriage	−0.001	0.091	−0.180	0.179	0.995
Children	0.925	0.270	0.391	1.460	0.001
Education	−0.075	0.090	−0.254	0.103	0.405
Subjective SES	0.021	0.076	−0.129	0.171	0.784
Pet attachment	−0.113	0.076	−0.262	0.037	0.139
Model 3					
Age	−0.039	0.098	−0.233	0.154	0.688
Gender	−0.385	0.164	−0.710	−0.061	0.020
Marriage	−0.015	0.089	−00.192	0.161	0.864
Children	0.866	0.266	0.341	1.392	0.001
Education	−0.084	0.089	−0.260	0.091	0.343
Subjective SES	0.036	0.075	−0.111	0.184	0.626
Pet attachment	−0.092	0.075	−0.239	0.056	0.222
Subjective SES × Pet attachment	−0.188	0.072	−0.330	−0.047	0.009

**Table 4:** Summary of the hierarchical multiple regression (*N* = 234).

Model	R	R Square	R Square Change	F Change	Sig. F Change
1	0.320	0.102	0.102	4.305	0.001
2	0.345	0.119	0.017	2.147	0.119
3	0.365	0.133	0.014	3.689	0.056

**Table 5:** Summary of the hierarchical regression model coefficients (*N* = 234).

	Standardized (β)	Std. Error	95% Confidence Interval		*p*
			Low	High	
Model 1					
Age	0.153	0.095	−0.034	0.339	0.108
Gender	−0.282	0.131	−0.540	−0.023	0.033
Marriage	0.064	0.088	−0.109	0.237	0.466
Children	0.251	0.262	−0.265	0.768	0.339
Pet ownership	0.037	0.141	−0.241	0.315	0.794
Objective SES	−0.043	0.078	−0.196	0.110	0.578
Model 2					
Age	0.158	0.094	−0.027	0.344	0.094
Gender	−0.283	0.131	−0.540	−0.025	0.031
Marriage	0.073	0.087	−0.099	0.245	0.403
Children	0.227	0.261	−0.288	0.742	0.386
Pet ownership	0.057	0.141	−0.220	0.335	0.684
Objective SES	−0.023	0.080	−0.182	0.135	0.771
Subjective SES	−0.071	0.066	−0.200	0.059	0.284
Pet attachment	−0.210	0.123	−0.452	0.033	0.090
Model 3					
Age	0.163		−0.021	0.348	0.083
Gender	−0.249		−0.507	0.009	0.059
Marriage	0.086		−0.086	0.257	0.326
Children	0.203		−0.310	0.715	0.437
Pet ownership	0.032		−0.245	0.309	0.818
Objective SES	−0.008		−0.166	0.150	0.921
Subjective SES	0.052		−0.128	0.231	0.573
Pet attachment	−0.211		−0.452	0.030	0.087
Subjective SES × Pet attachment	−0.243		−0.493	0.006	0.056

## Data Availability

The data presented in this study are available upon request from the corresponding author. The data are not publicly available due to participants’ privacy.

## Appendix A

The Measure of Fertility Intention.

Please indicate how much you agree or disagree with the following statements (1 = completely disagree, 9 = completely agree):

1. I love kids.
2. I like to go to baby stores.
3. I want to have my own children.
4. I am willing to spend a lot of time and money to raise my children.
